# Regression of Glomerular and Tubulointerstitial Injuries by Dietary Salt Reduction with Combination Therapy of Angiotensin II Receptor Blocker and Calcium Channel Blocker in Dahl Salt-Sensitive Rats

**DOI:** 10.1371/journal.pone.0107853

**Published:** 2014-09-18

**Authors:** Kazi Rafiq, Akira Nishiyama, Yoshio Konishi, Takashi Morikawa, Chizuko Kitabayashi, Masakazu Kohno, Tsutomu Masaki, Hirohito Mori, Hiroyuki Kobori, Masahito Imanishi

**Affiliations:** 1 Department of Pharmacology, Faculty of Medicine, Kagawa University, Kagawa, Japan; 2 Division of Nephrology and Hypertension, Osaka City General Hospital, Osaka, Japan; 3 Department of Cardiorenal and Cerebrovascular Medicine, Faculty of Medicine, Kagawa University, Kagawa, Japan; 4 Department of Gastroenterology, Faculty of Medicine, Kagawa University, Kagawa, Japan; University Medical Center Utrecht, Netherlands

## Abstract

A growing body of evidence indicates that renal tissue injuries are reversible. We investigated whether dietary salt reduction with the combination therapy of angiotensin II type 1 receptor blocker (ARB) plus calcium channel blocker (CCB) reverses renal tissue injury in Dahl salt-sensitive (DSS) hypertensive rats. DSS rats were fed a high-salt diet (HS; 4% NaCl) for 4 weeks. Then, DSS rats were given one of the following for 10 weeks: HS diet; normal-salt diet (NS; 0.5% NaCl), NS + an ARB (olmesartan, 10 mg/kg/day), NS + a CCB (azelnidipine, 3 mg/kg/day), NS + olmesartan + azelnidipine or NS + hydralazine (50 mg/kg/day). Four weeks of treatment with HS diet induced hypertension, proteinuria, glomerular sclerosis and hypertrophy, glomerular podocyte injury, and tubulointerstitial fibrosis in DSS rats. A continued HS diet progressed hypertension, proteinuria and renal tissue injury, which was associated with inflammatory cell infiltration and increased proinflammatory cytokine mRNA levels, NADPH oxidase activity and NADPH oxidase-dependent superoxide production in the kidney. In contrast, switching to NS halted the progression of hypertension, renal glomerular and tubular injuries. Dietary salt reduction with ARB or with CCB treatment further reduced blood pressure and partially reversed renal tissues injury. Furthermore, dietary salt reduction with the combination of ARB plus CCB elicited a strong recovery from HS-induced renal tissue injury including the attenuation of inflammation and oxidative stress. These data support the hypothesis that dietary salt reduction with combination therapy of an ARB plus CCB restores glomerular and tubulointerstitial injury in DSS rats.

## Introduction

Renal histopathological changes, once established, have been considered to be progressive and irreversible [1]. Indeed, glomerular and interstitial injuries are progressive in patients with chronic kidney disease [2]. Even if the primary cause is no longer present, time-dependent progression has also been reported [3]. However, a growing body of evidence has also indicated that glomerular and interstitial injuries can be reversible [4]–[9]. This possibility was first reported by Fioretto *et al*. [10], who showed regression of glomerular sclerosis in patients with diabetic nephropathy 10 years after pancreatic transplantation. Reversal of renal tissue lesions was also seen in patients with IgA glomerulonephritis [7], as well as in experimental animal models [4]–[6]. Thus, the new demand upon therapeutic intervention in renal disease is not only the halting of progression, but also the restoration of injured renal tissue.

Regression of glomerular lesions has been indicated in subtotal nephrectomized rats receiving high doses of angiotensin-converting enzyme (ACE) inhibitors or angiotensin receptor blockers (ARBs) [5], [6]. These results were further confirmed by Adamczak *et al* [11], who showed partial reversal of glomerulosclerosis after treatment with enalapril in subtotal nephrectomized rats. We have recently shown that treatment with an ARB regressed superficial glomerular podocyte injury in type 2 diabetic rats with overt albuminuria [12]. Thus, it is possible that continuous suppression of the renin-angiotensin system (RAS) can not only prevent, but also restore renal tissue injury. In this regard, recent experimental and clinical studies highlighted the potential beneficial effects of combination therapy with an ARB and calcium channel blocker (CCB) [13]–[18]. However, the renoprotective effect of dietary salt reduction with combined ARB plus CCB therapy during the development of salt-dependent hypertension has not yet been investigated.

The present study was designed to investigate whether dietary salt reduction with the combination therapy of ARB plus CCB reverses established glomerular and tubulointerstitial injuries in Dahl salt-sensitive (DSS) rats [19]. For this purpose, we examined the effects of dietary salt reduction with an ARB, olmesartan, plus a CCB, azelnidipine, in DSS rats with existing renal glomerular and tubular injuries induced by 4 weeks of treatment with a high-salt diet.

## Materials and Methods

### Animals

All experimental procedures were performed according to the guidelines for the care and use of animals established by Kagawa University, Kagawa, Japan. The experiments were approved by the Animal Experimentation Ethics Committee at Kagawa University. Male 5-week-old DSS rats (Seac Yoshitomi, Fukuoka, Japan) were fed standard rat chow for 1 week. All animals were maintained in a pathogen-free facility at a controlled temperature (24±2°C) and humidity (55±5%) with a 12-hour light/dark cycle. At the end of each experiment, organs were dissected under sodium pentobarbital anesthesia (65 mg/kg, i.p).

### Experimental Protocols

#### Protocol-1

At 6 weeks of age, DSS rats were selected at random to receive rat chow containing normal salt (NS: 0.5% NaCl, Oriental Yeast, Tokyo, Japan) or high salt (HS: 4% NaCl, Oriental Yeast) for 14 weeks from 6 to 20 weeks of age. From 6 to 20 weeks of age, HS-fed DSS rats were further treated with either vehicle (0.5% methyl cellulose, Nacalai Tesque, Kyoto, Japan), olmesartan (OLM: oral gavage; 10 mg/kg body weight/day, p.o.; Daiichi-Sankyo Co., Ltd., Tokyo, Japan), azelnidipine (AZEL: 3 mg/kg body weight/day, p.o.; Daiichi-Sankyo Co., Ltd.), their combination, or hydralazine (HYD: 50 mg/kg body weight/day, p.o.; Wako Co., Ltd., Osaka, Japan). The doses of OLM and AZEL were chosen on the basis of results from previous rat studies [12], [20]. Preliminary studies also showed that HYD at 50 mg/kg substantially decreased blood pressure in HS-treated DSS rats (data not shown). Kidneys were perfused with saline and harvested under sodium pentobarbital anesthesia (65 mg/kg) at 10, 15 and 20 weeks of age (n = 7 for each). All data from Protocol-1 are shown as in Supplemental Figures.

#### Protocol-2

Results in Protocol-1 showed that renal glomerular and tubular injuries were apparent after 4 weeks of treatment (from 6 to 10 weeks of age) with HS diet in DSS rats. Protocol-2 was performed to evaluate the potential reversal effects of dietary salt reduction with combination therapy of OLM plus AZEL on renal injury. “Dietary salt reduction” adopted in this study consisted simply of returning dietary salt content to normal levels (0.5% NaCl). Similar to Protocol-1, male 6-week-old DSS rats were fed either NS or HS diet for 4 weeks. Thereafter, HS diet was maintained or switched to NS diet and maintained for further 10 weeks until 20 weeks of age. Simultaneously, NS diet-treated DSS rats were treated with vehicle, OLM (10 mg/kg body weight/day, p.o.), AZEL (3 mg/kg body weight/day, p.o.), their combination or HYD (50 mg/kg body weight/day, p.o). Kidneys were perfused with saline and harvested at 10, 15 and 20 weeks of age (n = 7 for each) under sodium pentobarbital anesthesia (65 mg/kg).

### Blood Pressure Measurement

Systolic blood pressure (SBP) was monitored in conscious rats by tail-cuff plethysmography (BP-98A; Softron Co., Tokyo, Japan). Conscious rats were placed in a plastic holder resting on a warm pad maintained at 37°C during the measurement. Average readings were taken from consecutive five measurements for each rat after the rats had become acclimated to the environment [21].

### Urine Collection and Analyses

Twenty-four-hour urine samples were collected using metabolic cages. Urine samples were stored at −20°C after centrifugation until analysis. Urine protein excretion was determined using a protein assay kit (MicroTP-test, Wako Co. Ltd., Osaka, Japan) [22].

### Sample Preparation

Kidney tissues were cut and fixed in 10% buffered paraformaldehyde or embedded in Tissue-Tek OCT compound (Sakura Finetech Japan Co., Ltd., Tokyo, Japan), and remaining tissues were snap-frozen in liquid nitrogen. Small samples of renal cortical tissue were also collected in RNAlater and kept at 4°C overnight. RNAlater-treated samples were then snap-frozen in liquid nitrogen and stored at −80°C until processing for RNA extraction and reverse transcription-polymerase chain reaction (RT-PCR) analysis.

### Real time RT-PCR

Gene expression in renal cortical tissue was analyzed by RT-PCR using a LightCycler FastStart DNA Master SYBR Green I kit and an ABI Prism 7000 Sequence Detection System (Applied Biosystems, Foster City, USA) as described previously [18], [23]. The oligonucleotide primer sequences for rat β-actin, TGF-β_1_, α-SMA, type 1 collagen, MCP-1, PAI-1, gp91phox, Nox-1, E-cadherin, and fibroblast-specific protein 1 (FSP-1) are listed in Table 1. All data are expressed as the relative difference to the 10-week values of the DSS + NS group, after normalization to β-actin expression.

**Table 1:** The oligonucleotide primer sequences for real-time RT-PCR.

Name		Sequences
β-actin	sense	5′- CCCTGGCTCCTAGCACCAT-3′
	antisense	5′- CCTGCTTGCTGATCCACATCT-3′
TGF-β	sense	5′- GCCTGAGTGGCTGTCTTTTGA-3′
	antisense	5′- GAAGCGAAAGCCCTGTATTCC-3′
MCP-1	sense	5′- CTCAGCCAGATGCAGTTAATGC-3′
	antisense	5′- GACACCTGCTGCTGGTGATTC-3′
PAI-1	sense	5′- GCCCTACCACGGCGAAACCC-3′
	antisense	5′- TGATGGCGGAGAGGGGCACA-3′
gp91phox	sense	5′- TGGTGATGTTAGTGGGAGC-3′
	antisense	5′-3′ CTTTCTTGCATCTGGGTCT
Nox-1	sense	5′- TGGACGAATTAGGCAAACCG-3′
	antisense	5′- TTGGGGTGGGCAGTAGCTAT-3′
α-SMA	sense	5′- ACGGCGGCTTCGTCTTCT-3′
	antisense	5′- CCAGCTGACTCCATGCCAAT-3′
type 1 collagen	sense	5′- TCACCTACAGCACGCTTG-3′
	antisense	5′- GGTCTGTTTCCAGGGTTG-3′
E-cadherin	sense	5′- GTGCCACCACCAAAGATA-3′
	antisense	5′- GGCTGAGACAACCCTAAT-3′
FSP-1(S100a4)	sense	5′- AGCACTTCCTCTCTCTTGGTC-3′
	antisense	5′- GCTCCTTGAGCTCTGTCTTGT-3′

TGF-β; transforming growth factor β, MCP-1; monocyte chemoattractant protein 1, PAI-1; plasminogen activator inhibitor 1, α-SMA; alpha smooth muscle actin, FSP-1; fibroblast-specific protein-1.

### Histological Analyses

Kidney tissues fixed with 10% paraformaldehyde were embedded in paraffin, sectioned into 3 µm-thick slices, and stained with periodic acid-Schiff (PAS) or Masson’s trichrome (MT) reagent to evaluate glomerular and tubulointerstitial injuries, respectively [23]. The percentage of PAS-positive area in glomeruli was measured using image analysis software, WinROOF (Mitani Co., Tokyo, Japan). A total of 40–45 glomeruli were examined for each rat and the average percentage of affected lesions was calculated for each rat. To evaluate glomerular hypertrophy (glomerular diameter), an area containing approximately 25–30 glomeruli per kidney section was measured with Image-Pro plus software (Media Cybernetics, Bethesda, MD, USA). Quantification of mononuclear cell infiltration in the interstitium was determined by counting mononuclear cells in 10 fields at ×400 magnification and expressed as cells/mm^2^. The extent of the interstitial fibrotic area was evaluated quantitatively by automatic image analysis, which determined the area occupied by interstitial tissue positive for MT-staining as described previously [18]. Glomerular podocyte injury was evaluated by immunohistochemistry for desmin and was performed, as previously described [18], [21]. All morphometric measurements were performed in a blinded manner to avoid any bias.

### Evaluation of Renal Tissue 4-Hydroxy-2-Nonenal (4-HNE) Expression

The expression of 4-HNE, a marker of oxidative stress [24], [25], in the kidney was examined by immunohistochemistry using a mouse monoclonal anti-4-HNE antibody (Japan Institute for the Control of Aging, Fukuroi, Japan), as previously described [24]–[26].

### NADPH Oxidase Activity

NADPH oxidase-derived superoxide anion generation was measured using lucigenin-enhanced chemiluminescence, as previously described [18].

### Statistical Analysis

The results are presented as the means ± S.E.M. Statistical comparisons of the differences between groups were performed using one-way or two-way analysis of variance combined with Newman–Keuls post-hoc test. A *P* value of *P*<0.05 was considered statistically significant.

## Results

### Systolic Blood Pressure and Body Weight

**Figure 1:**
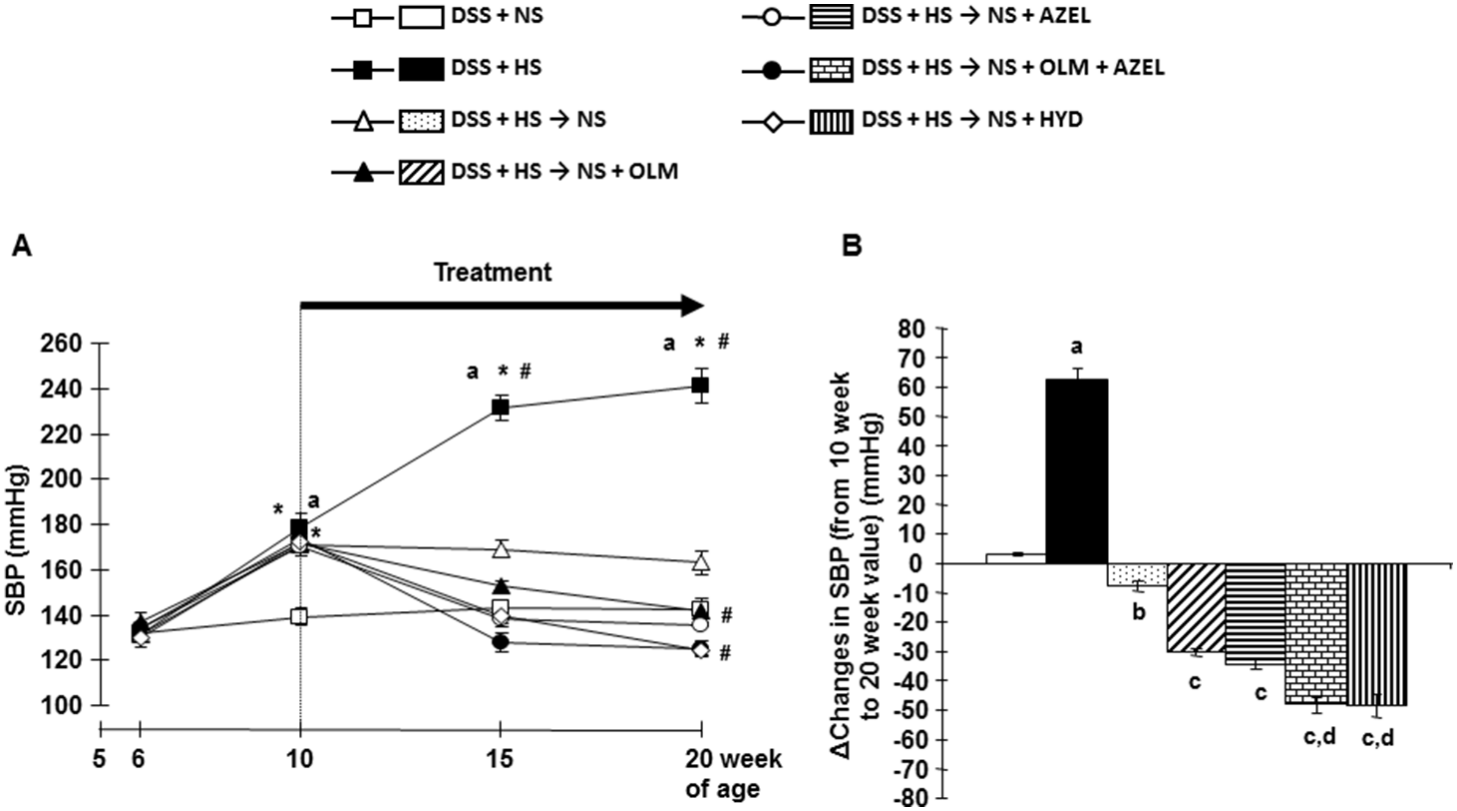
Systolic blood pressure (SBP) profile measured by tail-cuff plethysmography in Protocol2. **A**, SBP. Four weeks after high-salt (4%) loading (from 6 to 10 weeks of age), dietary salt reduction and/or drug treatment were given for 10 weeks (from 10 to 20 weeks of age). **B**, Delta changes in SBP were calculated by deducting 10-week values from 20-week values of the respective group. NS; normal salt, HS; high salt, OLM; olmesartan, AZEL; azelnidipine, HYD; hydralazine. *P<0.05 vs. baseline values of each respective group. ^#^ *P*<0.05 *vs.* 10-week values of each respective group or of DSS + HS group. ^a^ *P*<0.05 *vs.* DSS + NS, ^b^ *P*<0.05 *vs.* DSS + HS, ^c^ *P*<0.05 *vs.* DSS + HS → NS, ^d^ *P*<0.05 *vs.* DSS + HS → NS + OLM or DSS + HS → NS + AZEL.

Consumption of a HS diet for 4 weeks significantly increased SBP in DSS rats (Figure 1A, 1B and Figure S1). Further increases in SBP were observed time-dependently with the HS diet. Concomitant treatment with OLM or AZEL attenuated blood pressure elevation in HS-fed DSS rats (Figure S1). Greater blood pressure-lowering effects were observed in HS-fed DSS rats treated with a combination of OLM plus AZEL or HYD (Figure S1).

Four weeks after HS diet treatment, dietary salt reduction halted the progression of HS-induced increase in SBP (Figure 1A, 1B). Dietary salt reduction along with OLM, AZEL, combination of OLM plus AZEL or HYD showed lower SBP at 20 weeks of age, as compared with the values before treatment at 10 weeks of age (Figure 1A, 1B). During the 14-week treatment period, all rats showed continuous body weight gain and there were no significant differences among the groups (data not shown).

### Urine Protein Excretion

**Figure 2:**
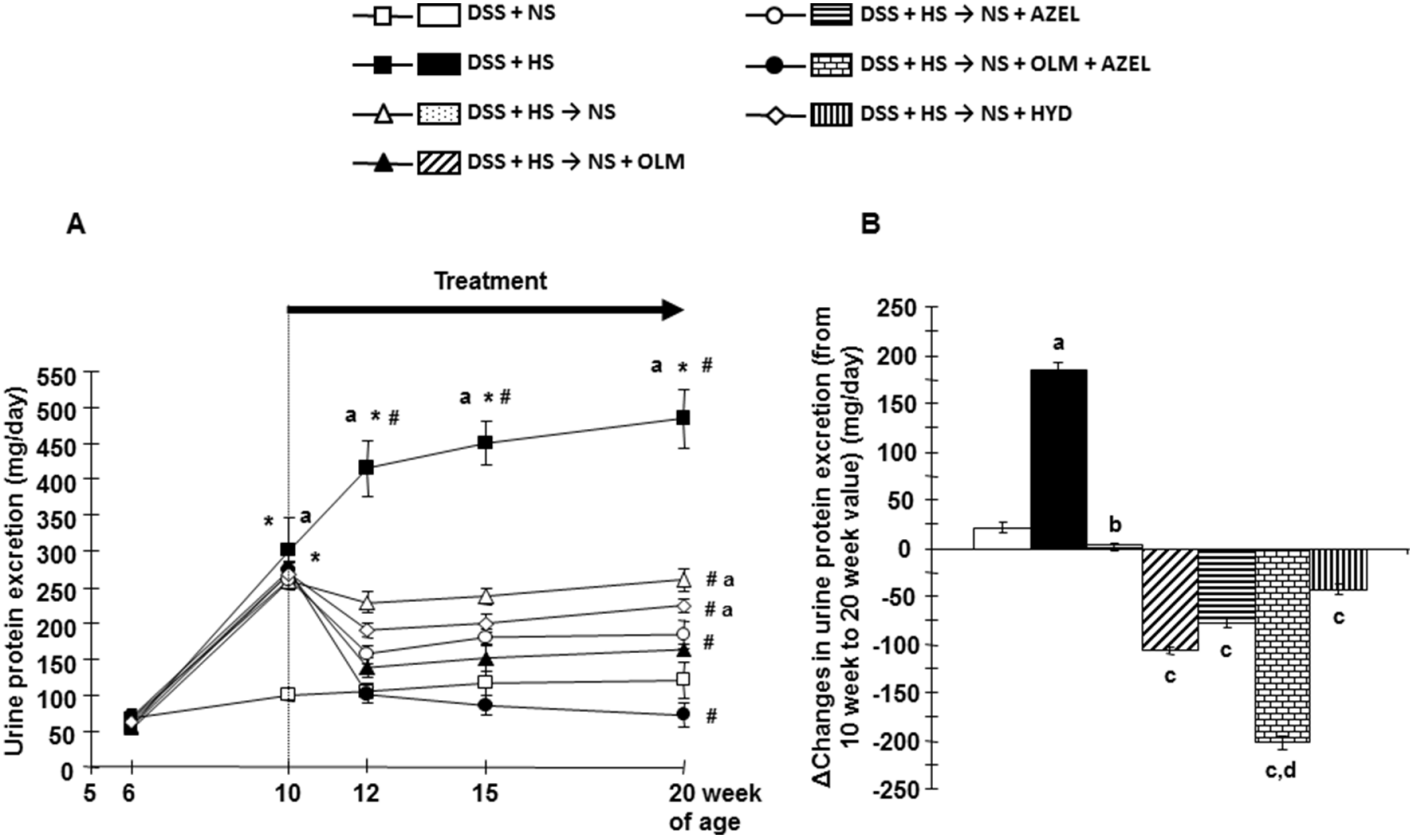
Urine protein excretion profile in Protocol 2. **A**, Twenty-four-hour urine protein excretion. **B**, Delta changes in urine protein excretion were calculated by deducting 10-week values from 20-week values of each respective group. *P<0.05 *vs.* baseline values of each respective group. ^#^ *P*<0.05 *vs.* 10-week values of each respective group or of the DSS + HS group. ^a^ *P*<0.05 *vs.* DSS + NS, ^b^ *P*<0.05 *vs.* DSS + HS, ^c^ *P*<0.05 *vs.* DSS + HS → NS, ^d^ *P*<0.05 *vs.* DSS + HS → NS + OLM or DSS + HS → NS + AZEL.

Figure 2A, 2B and Figure S2 show that consumption of a HS diet for 4 weeks significantly increased urinary protein excretion in DSS rats. Further marked increases in urinary protein excretion were observed time-dependently with continued HS diet feeding. Concomitant treatment with OLM and AZEL, but not HYD, significantly attenuated the increase in urinary protein excretion in HS-fed DSS rats (Figure S2). Greater attenuation in the development of proteinuria was observed in HS-fed DSS rats treated with the combination of OLM plus AZEL (Figure S2).

Four weeks after HS diet treatment, dietary salt reduction halted the progression of HS-induced increase in urinary protein excretion (Figure 2A, 2B). Dietary salt reduction with OLM, AZEL or HYD administration significantly reduced HS-induced increase in urinary protein excretion. Dietary salt reduction with the combination of OLM plus AZEL further decreased urinary protein excretion (Figure 2A, 2B).

### Renal Glomerular and Tubulointerstitial Injuries

**Figure 3:**
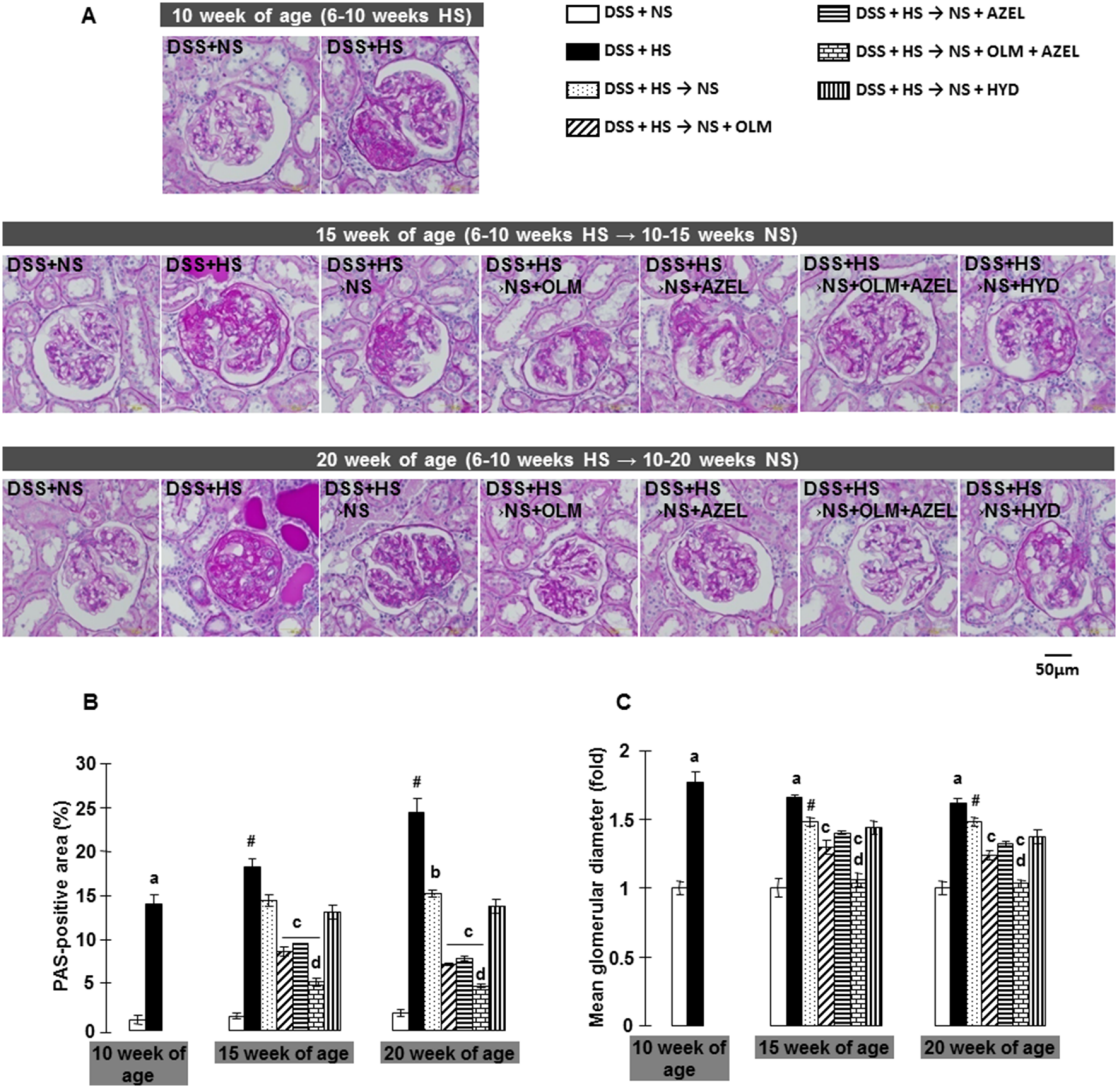
Renal histopathological changes were evaluated by examining periodic acid-Schiff (PAS) staining in Protocol 2. **A**, Representative images of PAS-stained renal sections (scale bar shows the values). **B**, The PAS-positive area within total glomerular area. **C**, Mean glomerular diameters expressed as fold changes to 10-week values of the DSS+NS group. ^#^ *P*<0.05 *vs.* 10-week values of DSS + HS group. ^a^ *P*<0.05 *vs.* DSS + NS, ^b^ *P*<0.05 *vs.* DSS + HS, ^c^ *P*<0.05 *vs.* DSS + HS → NS, ^d^ *P*<0.05 *vs.* DSS + HS → NS + OLM or DSS + HS → NS + AZEL.

The morphology of renal tissue injury was investigated by PAS staining (Figure 3A, 3B, 3C and Figure S3A, S3B). In DSS rats, consumption of a HS diet for 4 weeks induced glomerular sclerosis, as assessed by an increase in PAS-positive area within the glomeruli. Glomerular hypertrophy, tubular dilatation and extensive protein cast formation were also observed and progressed in a time-dependent manner during the experimental period. In protocol-1, concomitant treatment with OLM and AZEL, but not with HYD, significantly attenuated HS-induced renal histological changes. Furthermore, the combination of OLM plus AZEL treatment elicited a greater renoprotective effect against HS-induced renal tissue injuries (Figure S3A, S3B).

In protocol-2, rats fed a HS diet for 4 weeks also exhibited glomerular sclerosis and hypertrophy, tubular dilatation and extensive protein cast formation, all of which were halted by dietary salt reduction (Figure 3A, 3B, 3C). Dietary salt reduction in conjunction with OLM or with AZEL, but not with HYD, attenuated HS-induced renal tissue injuries. In contrast, dietary salt reduction with the combination of OLM plus AZEL significantly reversed HS diet-induced renal tissue injuries, although dilated tubules and injured glomeruli did not fully recover (Figure 3A–3C).

**Figure 4:**
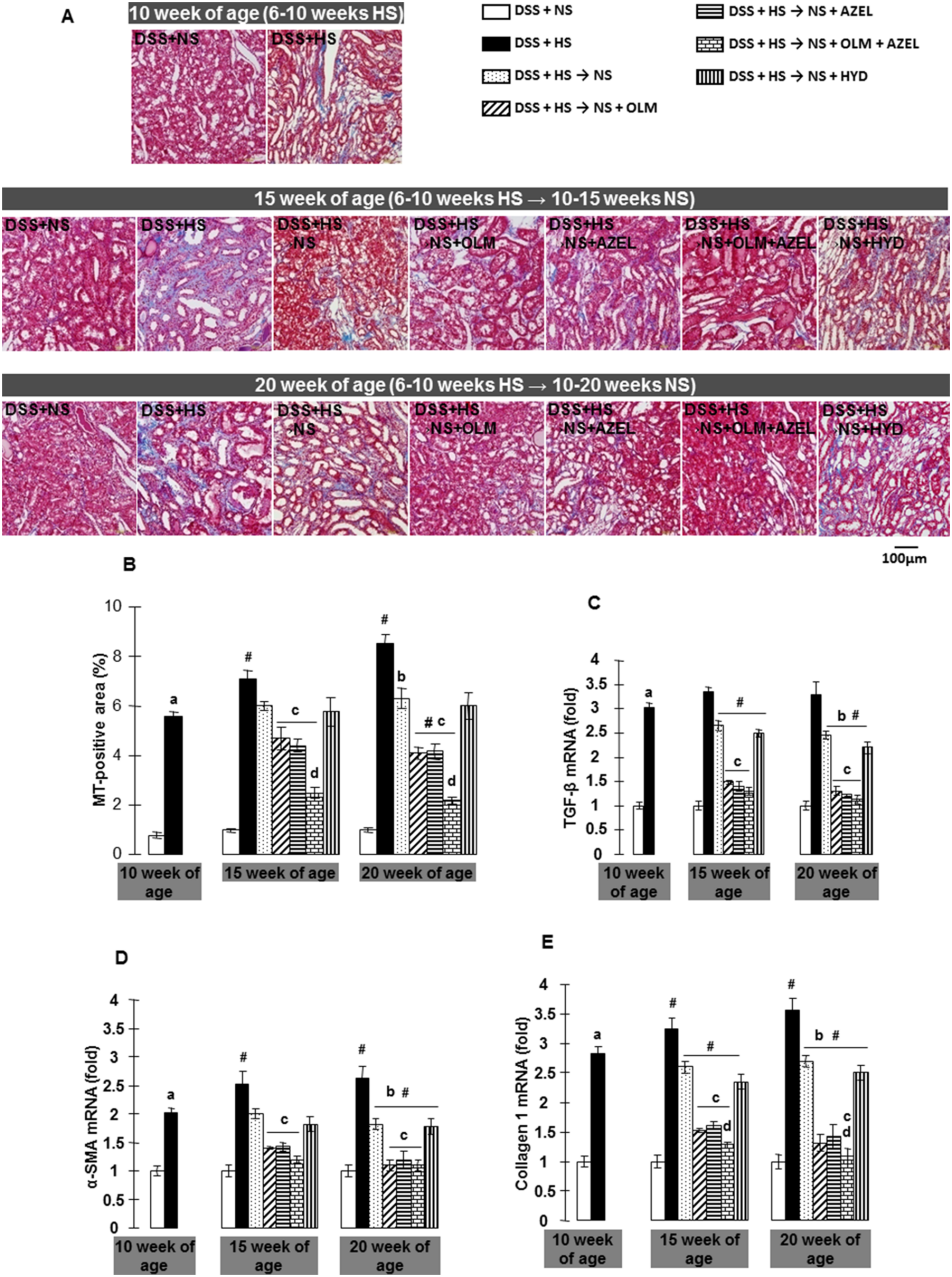
Renal tubulointerstitial fibrosis was detected by Masson’s trichrome (MT) staining in Protocol 2. **A**, Representative micrographs of MT-stained renal sections (scale bar shows the values). **B**, Quantitative analysis of MT-positive area. **C**, mRNA expression of transforming growth factor β (TGF-β), **D**, α-smooth muscle actin (α-SMA), and **E**, type 1collagen were quantitatively analyzed by real time RT-PCR. β-actin was used as an internal control and results are expressed as fold changes to 10-week values of the DSS + NS group. ^#^ *P*<0.05 *vs.* 10-week values of the DSS + HS group. ^a^ *P*<0.05 *vs.* DSS + NS, ^b^ *P*<0.05 *vs.* DSS + HS, ^c^ *P*<0.05 *vs.* DSS + HS → NS, ^d^ *P*<0.05 *vs.* DSS + HS → NS + OLM or DSS + HS → NS + AZEL.

DSS rats fed a HS diet for 4 weeks showed significant tubulointerstial fibrosis as assessed by quantification of the MT-positive area (Figure 4A, 4B and Figure S4A, S4B). HS-induced tubulointerstitial fibrosis progressed in a time-dependent manner during the experimental period. In contrast, concomitant treatment with OLM or AZEL, but not with HYD, significantly attenuated HS-induced tubulointerstitial fibrosis (Figure S4A, S4B). Furthermore, a greater protective effect against tubulointerstitial fibrosis was elicited by the combination of OLM plus AZEL treatment.

Four weeks after treatment with HS diet, dietary salt reduction halted the progression of HS-induced tubulointerstitial fibrosis (Figure 4A, 4B), and reduced the expression of profibrotic markers, such as TGF-β, α-SMA, and type 1 collage in renal cortical tissue. Dietary salt reduction with OLM or AZEL, but not with HYD, further attenuated HS-induced profibrotic gene expression in renal cortical tissue and also tubulointerstitial fibrosis. Furthermore, dietary salt reduction with the combination of OLM plus AZEL showed greater inhibition of elevated renal profibrotic gene expression and tubulointerstitial fibrosis (Figure 4A, 4B).

To demonstrate epithelial-mesenchymal transition (EMT) in the kidney, we measured the expression of a phenotypic epithelial cell marker, E-cadherin, and a phenotypic myofibroblast cell marker, FSP-1. DSS rats fed a HS diet for 4 weeks showed decreased expression of the E-cadherin gene and increased expression of the mesenchymal marker FSP-1 in renal cortical tissues (Table 2). Four weeks after treatment with HS diet, dietary salt reduction alone and with OLM or AZEL, but not with HYD increased E-cadherin and decreased FSP-1 expression. Furthermore, dietary salt reduction with the combination of OLM plus AZEL further increased E-cadherin and decreased FSP-1 expression. These findings support the concept that cellular phenotype trans-differentiation occurs during the development of salt-dependent hypertension in rats (Table 2).

**Table 2:** Genes expression from protocol 2 (fold changes).

Groups	10 week of age				20 week of age			
	E-cadherin	FSP-1	Sgk-1	NHE-1	E-cadherin	FSP-1	Sgk-1	NHE-1
DSS + NS	1.00±0.04	1.00±0.06	1.00±0.1	1.00±0.09	1.00±0.02	1.00±0.12	1.00±0.12	1.00±0.0
DSS + HS	0.52±0.30_a_	2.42±0.15_a_	2.33±0.11_a_	1.99±0.04_a_	0.41±0.02_a_	3.15±0.24_a_,_#_	2.99±0.10_a_	2.10±0.09_a_
DSS + HS → NS	-	-	-	-	0.48±0.03_b_	2.33±0.06_b_	2.38±0.11	1.80±0.17
DSS + HS → NS + OLM	-	-	-	-	0.73±0.05_c_	1.37±0.09_c_	1.37±0.05_c_	1.25±0.06_c_
DSS + HS → NS + AZEL	-	-	-	-	0.6±0.04	1.50±0.49_c_	1.35±0.79_c_	1.26±0.08_c_
DSS + HS → NS + OLM + AZEL	-	-	-	-	0.95±0.02_c,d_	1.10±0.02_c,d_	1.03±0.08_c,d_	0.99±0.25_c_
DSS + HS → NS + HYD	-	-	-	-	0.52±0.06	2.10±0.22	2.18±0.16	1.73±0.10

FSP-1; fibroblast-specific protein-1, Sgk-1; serum and glucocorticoid-regulated kinases-1, NHE-1; Na^+^/H^+^ exchanger isoform-1, DSS; Dahl salt-sensitive rats, NS; normal salt (0.5% NaCl), HS; High salt (4% NaCl), OLM; olmesartan, AZEL; azelnidipine, HYD; hydralazine. β-actin was used as an internal control and results are expressed as fold changes to 10-week values of the DSS + NS group. _#_ *P*<0.05 *vs.* 10-week values of the DSS + HS group. _a_ *P*<0.05 *vs.* DSS + NS, _b_ *P*<0.05 *vs.* DSS + HS, _c_ *P*<0.05 *vs.* DSS + HS → NS, _d_ *P*<0.05 *vs.* DSS + HS → NS + OLM or DSS + HS → NS + AZEL.

**Figure 5:**
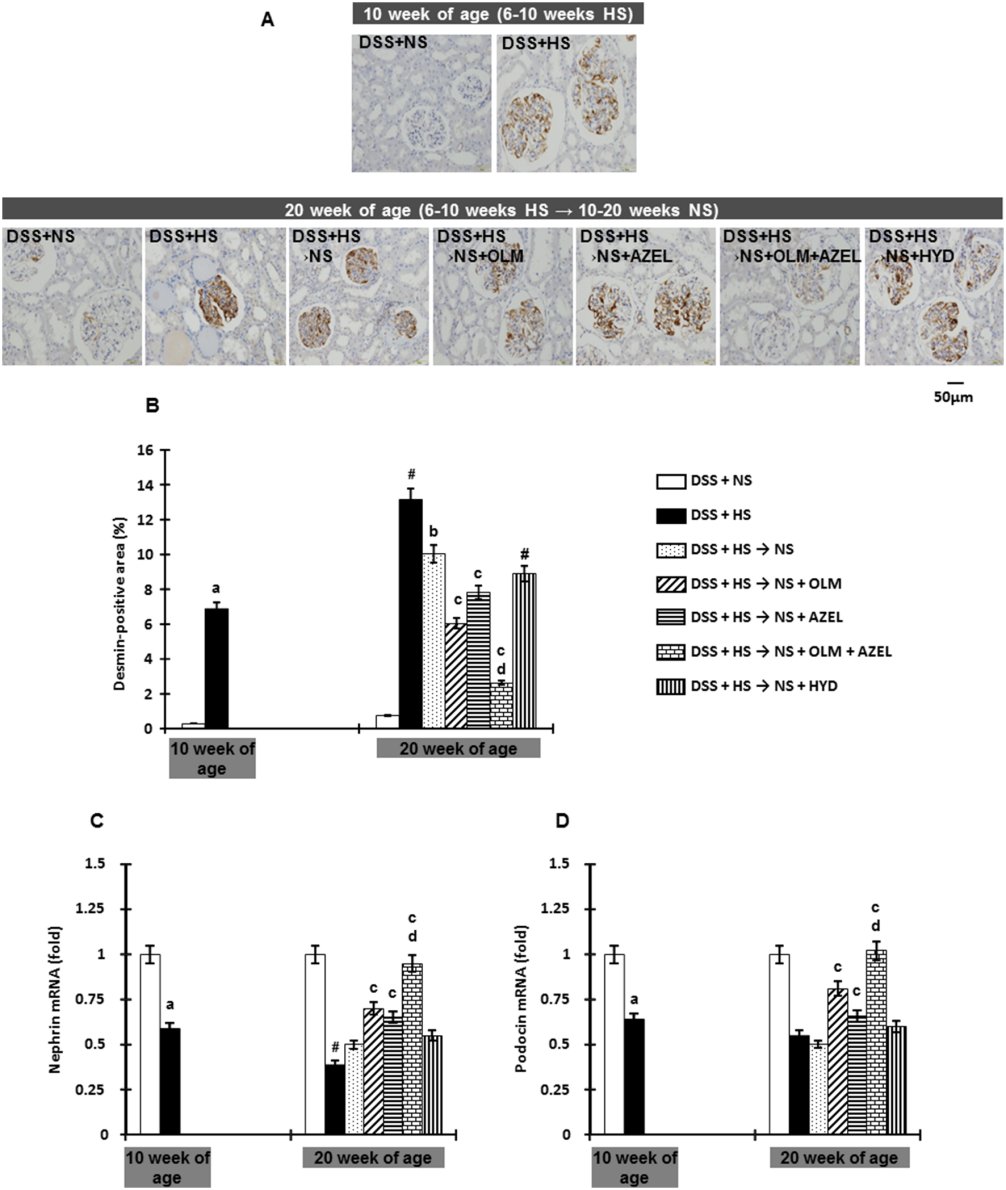
Glomerular podocyte injury was detected by immunohistochemistry for Desmin in Protocol 2. **A**, Representative micrographs of Desmin-stained renal sections (scale bar shows the values). **B**, Quantitative analysis of Desmin-positive area. **C**, mRNA expression of nephrin, and **D**, podocin were quantitatively analyzed by real time RT-PCR. β-actin was used as an internal control and results are expressed as fold changes to 10-week values of the DSS + NS group. ^#^ *P*<0.05 *vs.* 10-week values of the DSS + HS group. ^a^ *P*<0.05 *vs.* DSS + NS, ^b^ *P*<0.05 *vs.* DSS + HS, ^c^ *P*<0.05 *vs.* DSS + HS → NS, ^d^ *P*<0.05 *vs.* DSS + HS → NS + OLM or DSS + HS → NS + AZEL.

**Figure 6:**
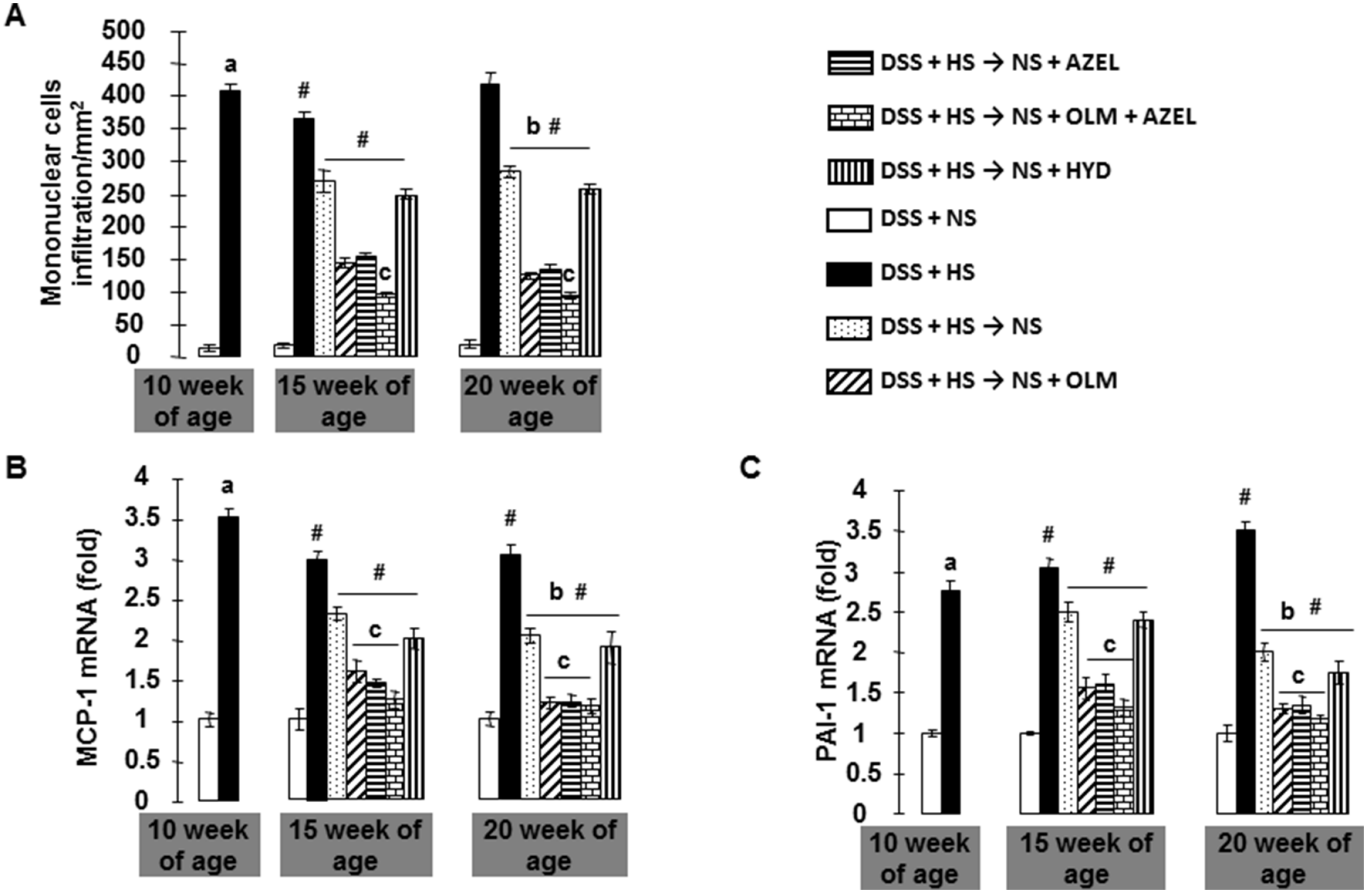
Cellular infiltration and proinflammatory cytokine mRNA levels in Protocol 2. **A**, The number of mononuclear cells in the interstitial space of the kidney was quantified. Renal cortical tissues (**B**) monocyte chemoattractant protein 1 (MCP-1), and (**C**) plasminogen activator inhibitor 1 (PAI-1) mRNA levels were determined by real time RT-PCR. β-actin was used as an internal control and results are expressed as fold changes to 10-week values of the DSS + NS group. ^#^ *P*<0.05 *vs.* 10-week values of DSS + HS group. ^a^ *P*<0.05 *vs.* DSS + NS, ^b^ *P*<0.05 *vs.* DSS + HS, ^c^ *P*<0.05 *vs.* DSS + HS → NS, ^d^ *P*<0.05 *vs.* DSS + HS → NS + OLM or DSS + HS → NS + AZEL.

Glomerular podocyte injury was determined by desmin expression as evaluated by immunohistochemistry [21], [27]. DSS rats fed a HS diet for 4 weeks showed a markedly increased glomerular desmin-positive area, which increased in a time-dependent manner over the experimental period (Figure 5A, 5B). We also measured the gene expression of nephrin and podocin, components of the slit diaphragm between adjacent podocytes. Renal cortical tissue nephrin and podocin mRNA levels were significantly lower in HS-diet fed rats than in NS-diet fed rats (Figure 5C and 5D). Four weeks after HS treatment, dietary salt reduction alone and with OLM or AZEL attenuated desmin expression. Furthermore, dietary salt reduction in combination with OLM plus AZEL reversed HS-induced glomerular podocyte injury (Figure 5A–6D).

### Renal Inflammation and Associated Genes Expression

In DSS rats fed a NS diet, few mononuclear cells were observed in the renal interstitial space (Figure 6A). However, DSS rats treated with a HS diet for 4 weeks had significant infiltration of mononuclear cells in the renal interstitial space, especially pericapillary and periglomerular areas (Figure 6A). These cellular infiltrations were associated with increased mRNA levels of proinflammatory cytokines, such as MCP-1 and PAI-1 in renal cortical tissue (Figure 6B–6D). Four weeks after HS diet treatment, dietary salt reduction attenuated HS-induced inflammatory cell accumulation and increases in mRNA levels of proinflammatory cytokines. Dietary salt reduction with OLM, AZEL or a combination of OLM plus AZEL, but not with HYD, significantly attenuated HS-induced increases in mRNA levels of proinflammatory cytokines and inflammatory cell accumulation (Figure 6A–6D).

### Renal Tissue Superoxide Anion Production, NADPH Oxidase Activity and NADPH Oxidase Subunits Gene Expression

**Figure 7:**
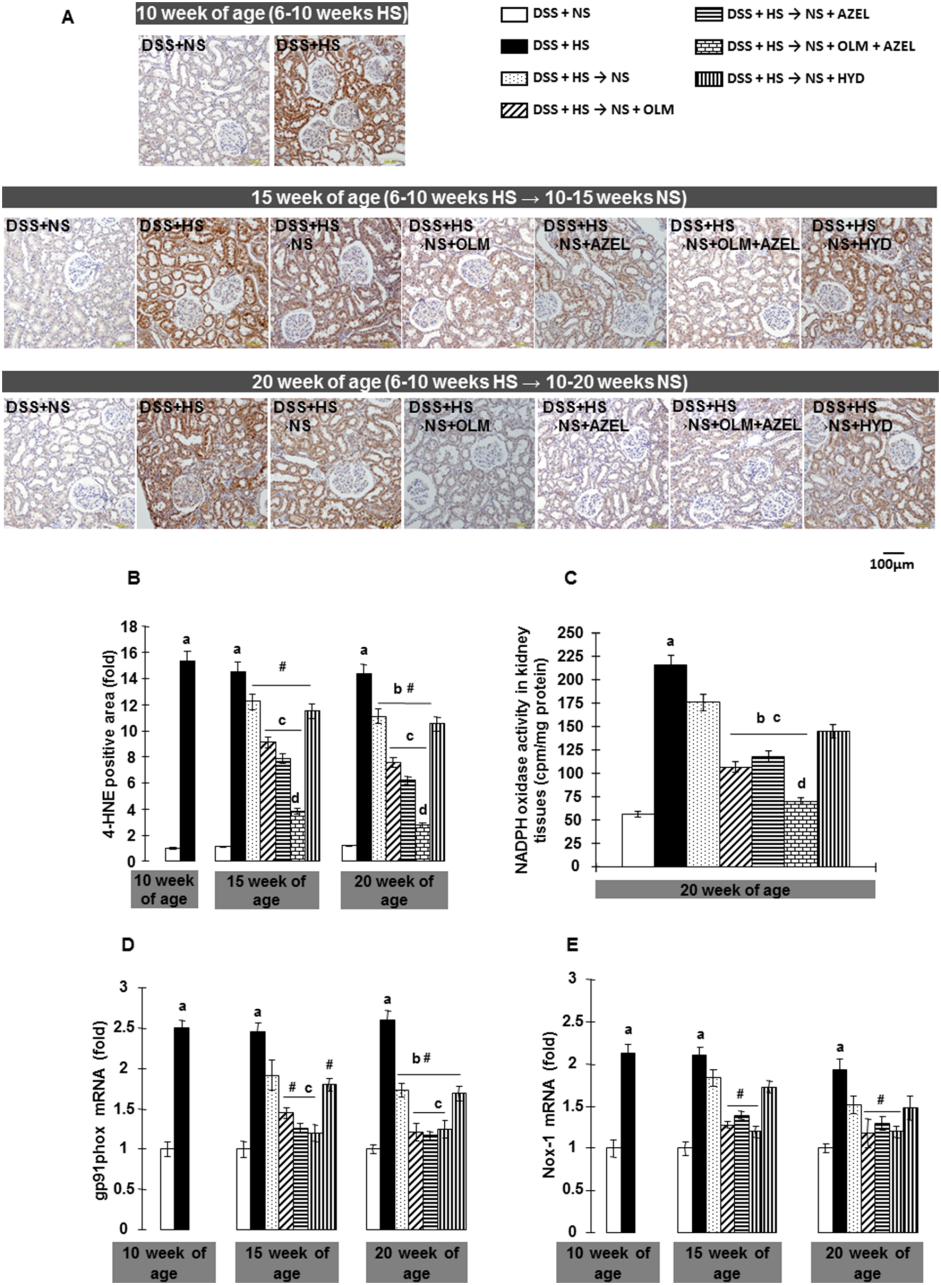
NADPH oxidase dependent superoxide anion production in renal tissue in Protocol 2. **A**, Representative images of 4-hydroxy-2-nonenal (4-HNE) immunostaining (scale bar shows the values). **B**, Quantitative analysis of 4-HNE-positive area. **C**, NADPH oxidase activity in homogenized renal cortical tissues measured by lucigenin-enhanced chemiluminescence. Renal cortical tissue (**D**) gp91phox and (**E**) Nox-1 mRNA levels were determined by real time RT-PCR. β-actin was used as an internal control and results are expressed as fold changes to 10-week values of the DSS + NS group. ^#^ *P*<0.05 *vs.* 10-week values of DSS + HS group. ^a^ *P*<0.05 *vs.* DSS + NS, ^b^ *P*<0.05 *vs.* DSS + HS, ^c^ *P*<0.05 *vs.* DSS + HS → NS, ^d^ *P*<0.05 *vs.* DSS + HS → NS + OLM or DSS + HS → NS + AZEL.

Renal tissue oxidative stress was evaluated by 4-HNE immunohistochemistry and NADPH oxidase activity. DSS rats fed a HS diet for 4 weeks showed markedly increased 4-HNE-positive areas in both glomeruli and tubules (Figure 7A, 7B). Increased 4-HNE staining was associated with an increase in NADPH oxidase activity (Figure 7C) and upregulation of the gene expression of NADPH oxidase subunits, such as Nox-1 and gp91phox, in renal cortical tissue (Figure 7D, 7E). Four weeks after HS treatment, dietary salt reduction attenuated the HS-induced oxidative stress and increases in NADPH oxidase activity and NADPH oxidase subunit gene expression in renal tissue. Dietary salt reduction with OLM or with AZEL further decreased these parameters. Furthermore, dietary salt reduction with the combination of OLM plus AZEL reversed HS-induced increases in these parameters (Figure 7A–7E).

### Renal Tissue Mineralocorticoid Receptor Target Genes Expression

HS diet for 4 weeks induced renal oxidative stress in DSS rats, which was associated with MR activation as demonstrated by significant up-regulation of the MR target gene, serum and glucocorticoid-regulated kinases-1 (Sgk-1) in renal cortical tissue (Table 2). As Sgk-1 is reported to respond to multiple stimuli [28], [29], we evaluated an additional MR target gene, Na+/H+ exchanger isoform-1 (NHE1), and observed that HS diet-fed rats also had increased renal NHE1 expression (Table 2).

Four weeks after HS treatment, dietary salt reduction did not suppress the HS-induced Sgk-1 and NHE-1 gene expression in renal tissue. However, dietary salt reduction plus OLM or AZEL suppressed Sgk-1 and NHE-1 gene expression. Furthermore, dietary salt reduction with the combination of OLM plus AZEL normalized HS-induced MR activation (Table 2).

## Discussion

Recent clinical studies, including the OSCAR study, highlight the potential beneficial effects of the combination therapy of ARB and CCB in various pathophysiological conditions [13]–[17]. Additionally, a recent study showed that ARB plus CCB has beneficial effects against cerebrovascular-renal injury in type 2 diabetic mice [18]. Consistent with previous studies [19], [30], the present study showed that in DSS rats, 4 weeks of treatment with HS diet caused hypertension, proteinuria, glomerular and tubulointerstitial damage. These parameters progressed time-dependently by continued feeding with a HS diet. To the best of our knowledge we demonstrate, for the first time, that dietary salt reduction with combination therapy of an ARB plus CCB is able to reverse established HS-induced glomerular and tubulointerstitial injuries in DSS rats, which suggests a new therapeutic renoprotective strategy for patients with salt-dependent hypertension and renal disease.

Oxidative stress is a major contributing factor to the pathophysiology of salt-induced hypertension and renal injury in DSS rats [19], [30], [31]. In these animals, the HS diet-induced increases in proinflammatory and profibrotic molecules [32] were associated with increases in renal NADPH activity and urinary H_2_O_2_ and 8-isoprostane excretion [33]. In agreement with previous studies [32], [33], the present study showed that HS diet administration to DSS rats resulted in a marked increase in oxidative stress markers, such as renal 4-HNE expression, NADPH oxidase activity and NADPH oxidase subunit mRNA levels, which were associated with renal tissue injury. We also showed that the renoprotective effects of dietary salt reduction in conjunction with an ARB plus CCB were accompanied by reduction in levels of renal oxidative stress. These data suggest that the strong renoprotective effects of dietary salt reduction with an ARB plus CCB are mediated by their antioxidative effects.

In agreement with previous findings [19], [34], [35], we showed that a HS diet caused a significant infiltration of mononuclear cells into the renal interstitium, which was associated with elevated gene expressions of proinflammatory cytokines such as MCP-1 and PAI-1. The present study further documented that these inflammatory changes were dramatically reversed by dietary salt reduction with combination therapy of an ARB plus CCB. Previous studies showed that treatment with high doses of enalapril normalized renal inflammatory cell infiltration in subtotally nephrectomized rats [11]. During the development of salt-sensitive hypertension, inhibition of inflammatory cell infiltration by dietary salt reduction with an ARB plus CCB may be able to attenuate the fibrogenic process, as activated macrophages secrete profibrotic molecules such as TGF-β_1_ [36], which plays a role in epithelial-mesenchymal transition, resulting in the appearance of interstitial myofibroblasts. These myofibroblasts express α-SMA and synthesize large amounts of extra cellular matrix proteins, including collagens [37]. In agreement with these observations, the present study demonstrated that HS diet-fed rats exhibited upregulation of α-SMA and collagen type 1 expression, both of which were suppressed by dietary salt reduction in combination with an ARB plus a CCB. These observations are in line with the concept that by decreasing PAI-1, dietary salt reduction with ARB plus CCB increases proteolytic activity in injured glomeruli and tubulointerstitium, permitting enhanced matrix degradation [6], [11]. EMT is a biologic process by which tubular cells lose their epithelial phenotype, including expression of E-cadherin, and acquire mesenchymal characteristics such as the expression of mesenchymal proteins, including FSP-1 and α-SMA [38]. EMT may be an adaptive response of epithelial cells after kidney injury and is an integral part of renal fibrogenesis. The major driving force behind EMT during the fibrogenic phase appears to be various profibrotic growth factors, including TGF-β1. Activated fibroblasts/myofibroblasts are the principal cells responsible for the accumulation of ECM and fibrosis under pathological conditions [39]. The loss of E-cadherin is considered to be an early change in EMT [40]. In this study, we found that DSS rats fed a HS diet for 4 weeks had significantly increased expression of EMT markers and tubulointerstitial fibrosis and were associated with upregulation of TGF-β1, which were normalized by dietary salt reduction in combination with an ARB plus a CCB. These data suggest that the inhibition of EMT is involved, at least in part, in the mechanism by which dietary salt reduction with ARB plus CCB attenuates renal fibrosis.

Previous studies showed that the ARB OLM suppresses salt-induced MR activation, progression of hypertension and renal dysfunction in DSS rats [41], [42], suggesting that the renoprotective effect of OLM may be partially mediated through decreased MR activity. Furthermore, ROS and angiotensin receptor stimulation may enhance MR activity [42]. Thus, during the development of salt-dependent hypertension, regional MR stimulates the renin angiotensin system, leading to the activation of a vicious cycle [43], [44].

In the present study, HYD partially attenuated HS-induced overt proteinuria, which would be explained by its substantial SBP lowering effect. However, HYD did not ameliorate renal histological injuries, inflammation or oxidative stress. These data suggest that dietary salt reduction with combination therapy of ARB and CCB exerts its beneficial renoprotective effects for the restoration or regression of HS-induced renal tissues injuries beyond their blood pressure-lowering ability [5], [6], [10], [11]. However, there are some limitations to the present study as we did not evaluate the blood pressure using a telemetry system. Therefore, we are unable to investigate the changes in dipping pattern of blood pressure. Further studies are needed to address such issues. It is important to note that some parameters measured in this study were not significantly changed by salt reduction with combination therapy of an ARB and CCB. This may be due to variation of the data between individuals and/or the limited number of animals. Alternatively, it is possible that the molecules investigated do not play a predominant role in the beneficial effects of salt reduction with combination therapy. Further studies are required to determine the specific role of each molecule in the renoprotective effects of salt reduction with combination therapy.

In conclusion, and in agreement with previous observations [5], [6], [11], [45], the findings of the present study further strengthens the recent exciting concept of reversal or regression of established glomerular and tubulointerstitial lesions by dietary salt reduction with combination therapy of an ARB plus CCB, through attenuation of renal oxidative stress and inflammation. Furthermore, these results suggest a novel therapeutic strategy for the restoration of damaged kidney tissue in the initial stages of salt-sensitive hypertension.

## Supporting Information

Figure S1 **Systolic blood pressure (SBP) in Protocol-1.** Consumption of a HS diet for 4 weeks significantly increased SBP in DSS rats. Further increases in SBP were observed time-dependently with a continued HS diet. Concomitant treatment with OLM or AZEL attenuated blood pressure elevation in HS-fed DSS rats. Greater blood pressure-lowering effects were observed in HS-fed DSS rats treated with the combination of OLM plus AZEL or HYD. NS; normal salt, HS; high salt, OLM; olmesartan, AZEL; azelnidipine, HYD; hydralazine. ^#^ *P*<0.05 *vs.* 10-week values of each respective group. ^a^ *P*<0.05 *vs.* DSS + NS, ^b^ *P*<0.05 *vs.* DSS + HS, ^d^ *P*<0.05 *vs.* DSS + HS + OLM or DSS + HS + AZEL.
(TIF)

Figure S2 **Urinary protein excretion profile in Protocol-1.** Consumption of a HS diet for 4 weeks significantly increased urinary protein excretion in DSS rats. Further marked increases in urinary protein excretion were observed time-dependently by a continued HS diet. Concomitant treatment with OLM and AZEL significantly attenuated the increase in urinary protein excretion in HS-fed DSS rats. Greater attenuation in the development of proteinuria was observed in HS-fed DSS rats treated with the combination of OLM plus AZEL. ^#^ *P*<0.05 vs. 10-week values of each respective group or of the DSS + HS group. ^a^ *P*<0.05 *vs.* DSS + NS, ^b^ *P*<0.05 *vs.* DSS + HS, ^d^ *P*<0.05 *vs.* DSS + HS + OLM or DSS + HS + AZEL.
(TIF)

Figure S3 **Renal histopathological changes in Protocol-1.** **A**, Representative images of periodic acid-Schiff (PAS)-stained renal sections (scale bar shows the values). **B**, The PAS-positive area within total glomerular area. In DSS rats, consumption of a HS diet for 4 weeks induced glomerular sclerosis, as assessed by an increase in PAS-positive area within the glomeruli. Glomerular hypertrophy, tubular dilatation and extensive protein cast formation were also observed and progressed in a time-dependent manner during the experimental period. Concomitant treatment with OLM and AZEL significantly attenuated HS-induced renal histological changes. Furthermore, the combination of OLM plus AZEL treatment elicited a greater renoprotective effect against HS-induced renal tissue injury. ^#^ *P*<0.05 *vs.* 10-week values of the DSS + HS group. ^a^ *P*<0.05 *vs.* DSS + NS, ^b^ *P*<0.05 *vs.* DSS + HS, ^d^ *P*<0.05 *vs.* DSS + HS + OLM or DSS + HS + AZEL.
(TIF)

Figure S4 **Renal tubulointerstitial fibrosis was detected by Masson’s trichrome (MT) staining in Protocol-1.** **A**, Representative micrographs of MT-stained renal sections (scale bar shows the values). **B**, Quantitative analysis of MT-positive area. Consumption of a HS diet for 4 weeks induced significant tubulointerstitial fibrosis in DSS rats as assessed by quantification of the MT-positive area. HS-induced tubulointerstitial fibrosis progressed in a time-dependent manner during the experimental period. In contrast, concomitant treatment with OLM or AZEL significantly attenuated HS-induced tubulointerstitial fibrosis. Furthermore, a greater protective effect against tubulointerstitial fibrosis was elicited by treatment with the combination of OLM plus AZEL. ^#^ *P*<0.05 *vs.* 10-week values of DSS + HS group. ^a^ *P*<0.05 *vs.* DSS + NS, ^b^ *P*<0.05 *vs.* DSS + HS, ^d^ *P*<0.05 *vs.* DSS + HS + OLM or DSS + HS + AZEL.
(TIF)
