# Supplementary figures and images for: Regression of Glomerular and Tubulointerstitial Injuries by Dietary Salt Reduction with Combination Therapy of Angiotensin II Receptor Blocker and Calcium Channel Blocker in Dahl Salt-Sensitive Rats
Source: PLoS One. 2014 Sep 18;9(9):e107853. doi: 10.1371/journal.pone.0107853 (PMC4169441; doi:10.1371/journal.pone.0107853)

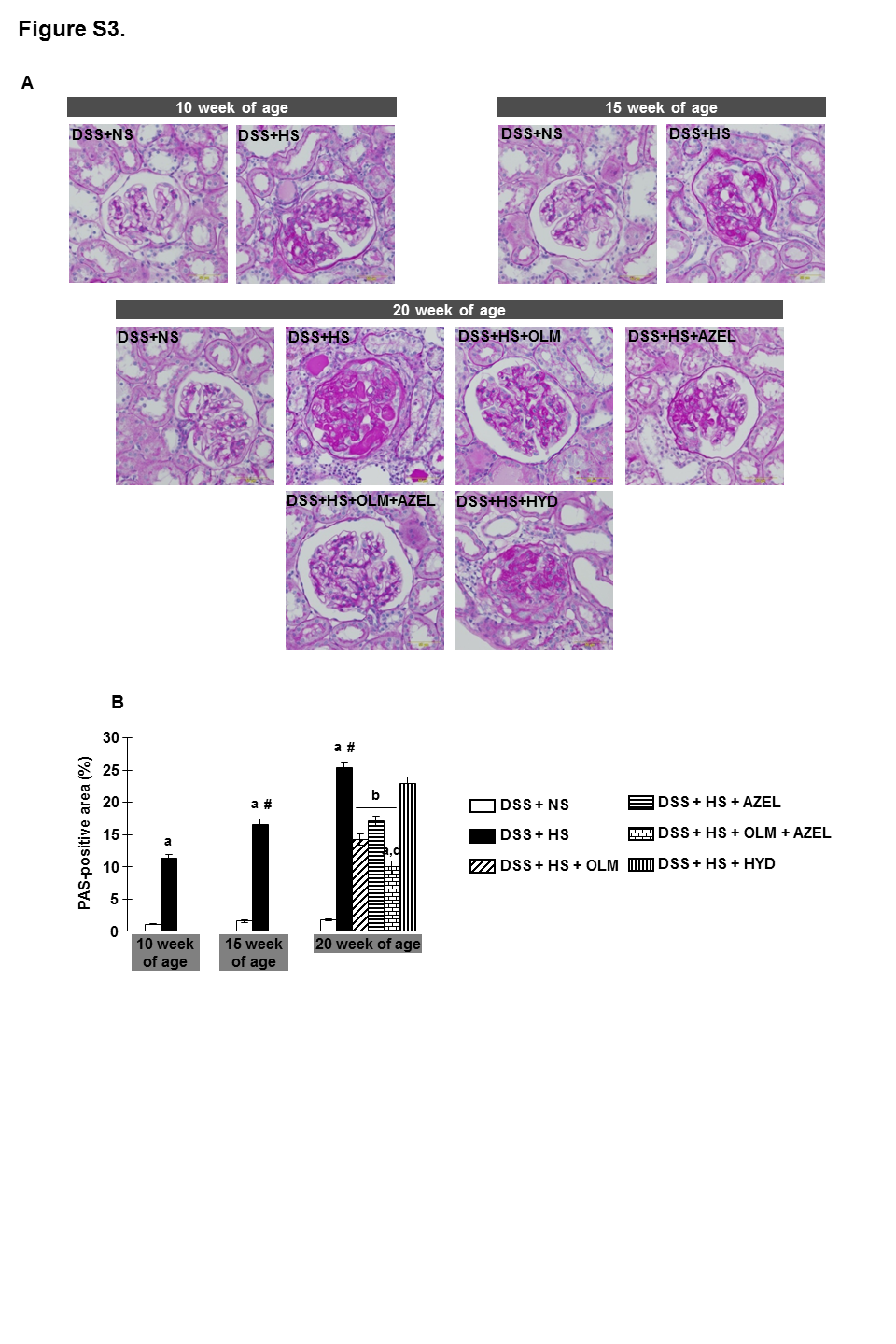

Supplement: Figure S3 — Renal histopathological changes in Protocol-1. A, Representative images of periodic acid-Schiff (PAS)-stained renal sections (scale bar shows the values). B, The PAS-positive area within total glomerular area. In DSS rats, consumption of a HS diet for 4 weeks induced glomerular sclerosis, as assessed by an increase in PAS-positive area within the glomeruli. Glomerular hypertrophy, tubular dilatation and extensive protein cast formation were also observed and progressed in a time-dependent manner during the experimental period. Concomitant treatment with OLM and AZEL significantly attenuated HS-induced renal histological changes. Furthermore, the combination of OLM plus AZEL treatment elicited a greater renoprotective effect against HS-induced renal tissue injury. # P<0.05 vs. 10-week values of the DSS + HS group. a P<0.05 vs. DSS + NS, b P<0.05 vs. DSS + HS, d P<0.05 vs. DSS + HS + OLM or DSS + HS + AZEL. (TIF) [file pone.0107853.s003.tif]

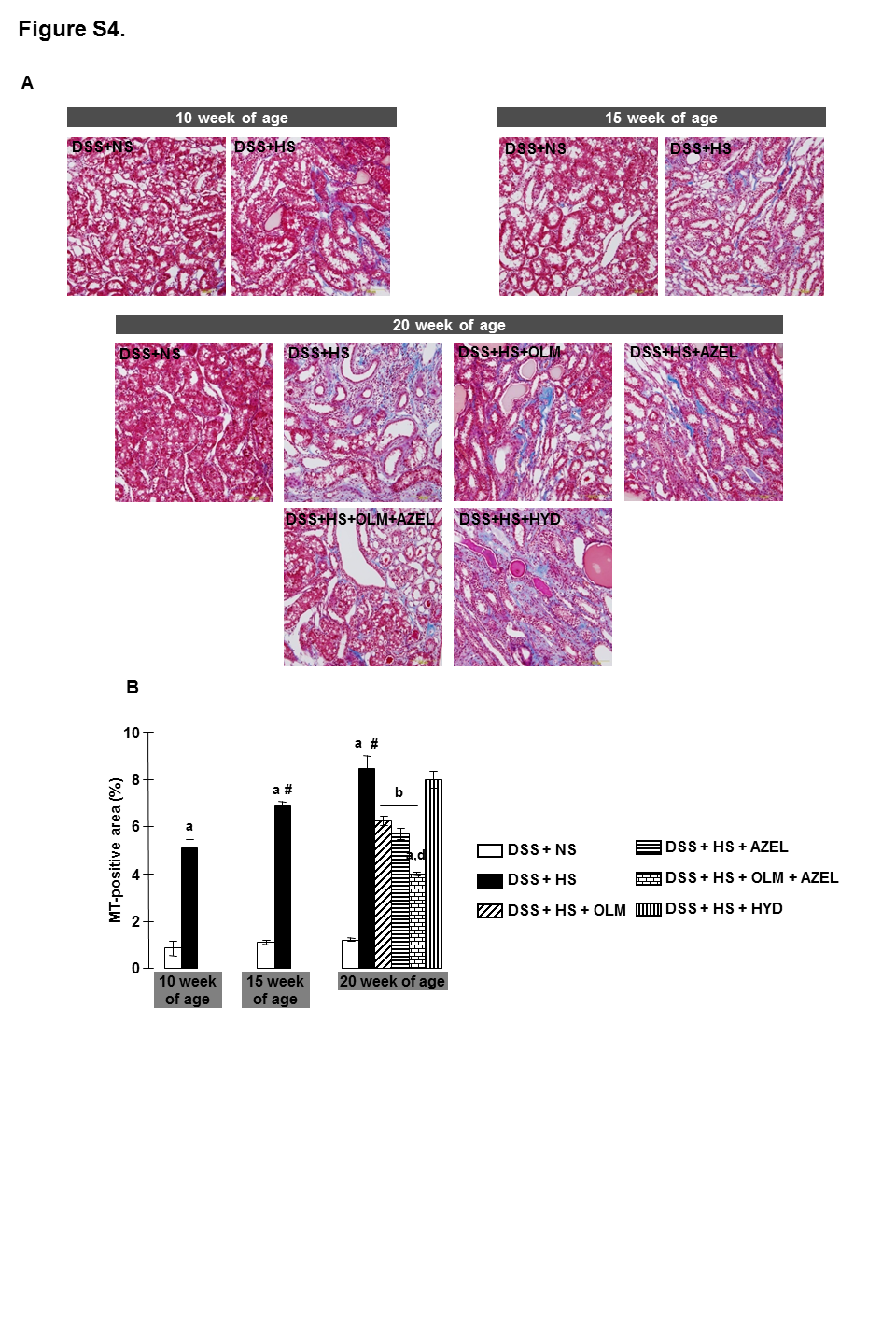

Supplement: Figure S4 — Renal tubulointerstitial fibrosis was detected by Masson’s trichrome (MT) staining in Protocol-1. A, Representative micrographs of MT-stained renal sections (scale bar shows the values). B, Quantitative analysis of MT-positive area. Consumption of a HS diet for 4 weeks induced significant tubulointerstitial fibrosis in DSS rats as assessed by quantification of the MT-positive area. HS-induced tubulointerstitial fibrosis progressed in a time-dependent manner during the experimental period. In contrast, concomitant treatment with OLM or AZEL significantly attenuated HS-induced tubulointerstitial fibrosis. Furthermore, a greater protective effect against tubulointerstitial fibrosis was elicited by treatment with the combination of OLM plus AZEL. # P<0.05 vs. 10-week values of DSS + HS group. a P<0.05 vs. DSS + NS, b P<0.05 vs. DSS + HS, d P<0.05 vs. DSS + HS + OLM or DSS + HS + AZEL. (TIF) [file pone.0107853.s004.tif]
